# Driving with Central Visual Field Loss II: How Scotomas above or below the Preferred Retinal Locus (PRL) Affect Hazard Detection in a Driving Simulator

**DOI:** 10.1371/journal.pone.0136517

**Published:** 2015-09-02

**Authors:** P. Matthew Bronstad, Amanda Albu, Alex R. Bowers, Robert Goldstein, Eli Peli

**Affiliations:** The Schepens Eye Research Institute, Massachusetts Eye and Ear, Harvard Medical School, Boston, MA, United States of America; Centre for Eye Research Australia, AUSTRALIA

## Abstract

We determined whether binocular central scotomas above or below the preferred retinal locus affect detection of hazards (pedestrians) approaching from the side. Seven participants with central field loss (CFL), and seven age-and sex-matched controls with normal vision (NV), each completed two sessions of 5 test drives (each approximately 10 minutes long) in a driving simulator. Participants pressed the horn when detecting pedestrians that appeared at one of four eccentricities (-14°, -4°, left, 4°, or 14°, right, relative to car heading). Pedestrians walked or ran towards the travel lane on a collision course with the participant’s vehicle, thus remaining in the same area of the visual field, assuming participant's steady forward gaze down the travel lane. Detection rates were nearly 100% for all participants. CFL participant reaction times were longer (median 2.27s, 95% CI 2.13 to 2.47) than NVs (median 1.17s, 95%CI 1.10 to 2.13; difference *p*<0.01), and CFL participants would have been unable to stop for 21% of pedestrians, compared with 3% for NV, *p*<0.001. Although the scotomas were not expected to obscure pedestrian hazards, gaze tracking revealed that scotomas did sometimes interfere with detection; late reactions usually occurred when pedestrians were entirely or partially obscured by the scotoma (time obscured correlated with reaction times, *r* = 0.57, *p*<0.001). We previously showed that scotomas lateral to the preferred retinal locus delay reaction times to a greater extent; however, taken together, the results of our studies suggest that any binocular CFL might negatively impact timely hazard detection while driving and should be a consideration when evaluating vision for driving.

## Introduction

Driving is the primary means of transportation in the U.S.[1] and driving cessation may reduce quality of life [2]. Many disorders, including AMD (Age-Related Macular Degeneration), cause binocular central field loss (CFL; a scotoma that includes the fovea) [3]. In the US, visual acuity is always considered in driver licensing but presence of central, or paracentral, scotomas is not (though it is considered in some countries including the UK[4] and Canada[5]). Patients may be unaware of their CFL[6] and drive despite their scotoma. However, older people with CFL frequently restrict their driving [7–10].

Verezen *et al*.[11] found that scotomas superior (25%) and inferior (11%) to the preferred retinal locus (PRL) were less common than scotomas lateral (50% right, 14% left) to the PRL in age-related macular degeneration (AMD) (we use visual field space to specify *PRL* and *scotoma location*). Vertical scotomas, however, are more common in juvenile macular degeneration (JMD); 70% of 30 eccentrically fixating JMD patients had scotomas above their PRL [12]. Because JMD affects younger, working-age people, with a greater need to drive, it is important to understand how vertical scotomas affect hazard detection during driving. Our simulator studies suggest that binocular paracentral lateral scotomas[13] and central scotomas lateral to the PRL[14] impair hazard detection when driving. We reported that CFL lateral (to the left or right) to the PRL markedly delayed reactions to pedestrian hazards approaching from the scotoma side [14]. Additionally, when hazards appeared outside the scotoma, responses were delayed (albeit less so), compared to normally sighted controls [14]. However, as highlighted in a recent editorial about that study [15], it remains to be determined whether CFL affects hazard detection when the field loss is vertical to the PRL.

Here we report driving simulator results from participants with vertical CFL: binocular scotomas above or below their PRL. Because pedestrian hazards typically appear on the side of the road and thus likely outside the vertical scotoma, we hypothesized that detection rates and reaction times would be similar for pedestrians approaching from the right or left. Because CFL participants use a non-foveal area for fixation, the retinal locations used to detect pedestrians are more eccentric than for controls; thus we predicted that their reaction times would be longer.

Drivers with CFL might compensate for their vision loss by scanning, *e*.*g*., to bring a hazard obscured by a scotoma into a seeing area of the visual field. However, results from our prior study suggest that many participants with lateral CFL did not compensate well by lateral scanning, as responses to the laterally-placed pedestrian hazards were often very delayed [14]. For drivers with vertical CFL, it is also possible that typical vertical gaze movements used when driving (*e*.*g*., to glance at the dashboard) might occasionally place on-road hazards, normally imaged on intact peripheral retina, into a scotoma and thereby delay detection. For example, a scotoma above the PRL may occlude a pedestrian when the driver looks down to the speedometer. We used gaze-tracking, and the position, size, and shape of each participant’s scotoma, to test this hypothesis.

## Materials and Methods

### Ethics Statement

The study followed the Declaration of Helsinki’s tenets and was approved by institutional review boards at Schepens Eye Research Institute and at the Veterans Administration Boston Healthcare System. Written informed consent was obtained from all participants.

### Participants

Participants with CFL were recruited from the Schepens and Veterans Administration hospital databases. We screened 38 CFL participants, 10 had vertical CFL and were enrolled; 28 had lateral CFL, of whom 11 completed the earlier study [14]. Seven of ten participants with vertical CFL completed this study (one withdrew due to simulator sickness, one did not meet visual acuity criteria, and one had scheduling conflicts). Seven age- (≤3yr. difference) and sex-matched normal vision controls, current drivers, were recruited from Schepens and the Harvard Cooperative Program on Aging. All participants passed (≤4 errors) the Short Portable Mental Status Questionnaire (SPMSQ),[16] and none were tested in the simulator previously. Table 1 summarizes the participants.

**Table 1:** Participant characteristics.

	Central Field Loss (CFL) (n = 7)	Normal Vision (NV)(n = 7)	Test of group differences
Current driver: n (%)	6 (86%)	7 (100%)	M-W *U* = 21, *p* = 0.71
Driving experience (years)*	49 ± 20 [15–68]	50 ± 20 [29–71]	*t*(12) = 0.24, *p* = 0.81
Male: n (%)	4 (57%)	4 (57%)	*p* > 0.99
Age (years)*	68 ± 18.15 [45–88]	68 ± 17.87 [46–87]	*t*(12) = 0.03, *p* = 0.98
SPMSQ*	11 ± 0.53 [10–11]	10 ± 0.98 [9–11]	*t*(12) = 0.34, *p* = 0.74
Binocular VA (logMAR)*	0.57 ± 0.28	0.01± 0.09	*t*(12) = 5.12, ***p* < 0.001**
[Snellen]	[20/32-20/200]	[20/16-20/25]	
Contrast Sensitivity (log)*	1.29 ± 0.33 [0.75–1.73]	1.78 ± 0.17 [1.73–1.95]	*t*(12) = 3.44, ***p* = 0.005**
Fixation stability* within 2°	73 ± 26 [31–100]	98 ± 4 [91–100]	*t*(10) = 2.17, *p* = 0.04
within 4°	94 ± 8 [81–100]	99 ± 3 [93–100]	*t*(10) = 1.26, *p* = 0.24
**CFL cause** AMD: n	3	n/a	n/a
Stargardt’s: n	3		
plaquenil toxicity: n	1		

* Mean ±Standard deviation [Range].

n/a = not applicable

### Vision Measures

**Fig 1:**
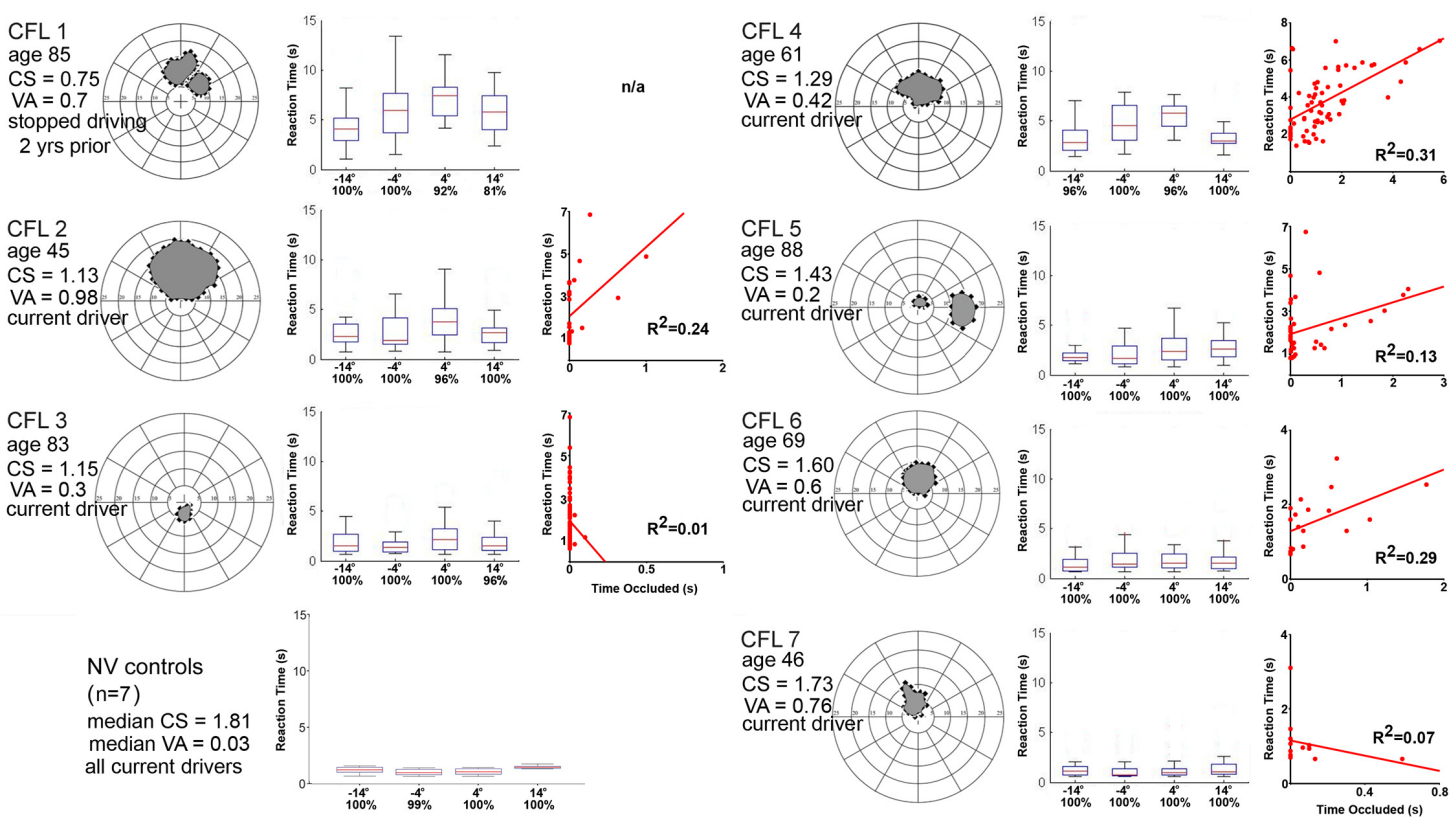
Binocular visual field plots (PRL at origin), Reaction Time (RT) boxplots for Central Field Loss (CFL) subjects (24–26 pedestrian appearances at each eccentricity), and individual plots of RT by scotoma occlusion time. RT boxplot for normally-sighted controls is at bottom left. Box lengths show the 25% to 75% interquartile range (IQR) and whiskers show the maximum extent of cases that are not outliers (values >1.5 times IQR). Detection rates for each eccentricity are below each plot. CFL participant age, binocular contrast sensitivity (CS), binocular visual acuity and driver status are to the left of the plots. CFL5 had a vertical central scotoma and a paracentral lateral scotoma from OS scotoma overlapping OD physiological blind spot.

CFL participants’ corrected binocular single letter visual acuity was 20/200 or better (Table 1), 20/25 or better for NV controls. All participants had at least 120° binocular field extent, measured with Goldmann perimetry (V4e target). CFL participants had a binocular central scotoma, six above and one below the binocular PRL (Fig 1). CFL5 also had a scotoma to the right of fixation resulting from overlap of the left eye monocular scotoma with the physiological blind spot of the right eye. People with only one eye all exhibit that scotoma yet they are permitted to drive in all jurisdictions. Central scotomas were mapped using a custom computerized test [17] (74 cd/m^2^ bright 0.74° square targets, 21 cd/m^2^ grey background, viewed binocularly from 1m, fixating a 1.23° cross). Standard binocular kinetic perimetry was used, measuring the scotoma from inside to out. Scotoma size was defined as the average diameter of four cardinal meridians through the center.

Contrast sensitivity (2.5° letters) was measured with a custom computer-based test that gives results consistent with the Pelli-Robson chart for a similar population (R. Woods, PhD, written communication, May 24, 2012). Monocular PRL locations were confirmed by fundus-tracking microperimeter (Nidek MP-1, Fremont, CA). Fixation stability was the percentage of fixation samples within 2° and 4° diameter while fixating a cross for 30 seconds.

### Driving Simulator

The simulator is a PP1000-x5 (FAAC Corp., Ann Arbor, MI), with five 60cm x 45cm CRTs (1024x768px, 60Hz, field of view 225° horizontal by 32° vertical). The simulator has automatic transmission and controls typical of American sedans. Data output is 30Hz, including handling, controls use, and locations of programmable entities in the virtual world. Additional details are available [18].

### Gaze Tracking

A video eyetracker (SmartEye Pro, SmartEye AB, Göteborg, Sweden) with 2 IR cameras recorded head and eye movements at 60Hz. Combined eye and head (gaze) data were integrated with simulator data; as simulator output was at 30Hz, every other SmartEye sample was analyzed. SmartEye claims gaze tracking accuracy of 0.5° over 90–110° rotation of gaze (www.smarteye.se). Using a conservative measure of gaze accuracy, the average of deviations from intended gaze point, we measured 0.86° at the monitor center and 4.7° at 35° laterally; however, hypothesis tests were performed on data collected within 15° of the monitor’s center.

### Procedure

Participants completed 10 test drives (each ~10min.) across 2–3 sessions. Test drives were on city roads or rural highways, 30mph and 60mph posted speed limits, respectively. Each drive included other traffic (mainly oncoming) and a variety of driving maneuvers (driving on straight and curved road segments in city and rural drives and right and left turns in the city drives, including responses to traffic lights and to traffic control signs). In addition, two drives included scenarios in which participants were required to overtake cars and two in which participants were asked to follow lead cars. Participants were instructed to obey normal traffic laws, drive as close to the speed limit as prudent and comfortable, and press the horn immediately after detecting pedestrians. Driving conditions were ideal for drivers with low vision; sunny, clear visibility and roads in good repair.

Pedestrians (2m tall, dressed in a grey top and trousers) appeared on either side of the road at one of four eccentricities (-14°, -4° on the left, 4°, 14° on the right relative to car heading) at 220’ (67m) distant in the city, 440’ (134m) on highways. Pedestrian appearances (n = 104) were evenly divided among the four eccentricities. Pedestrians walked or ran perpendicular to the participant’s car path on a collision course, as if to cross the road, but never crossed nor entered the car’s travel lane. Pedestrians appearing on the left were programmed not to co-occur with oncoming traffic. Pedestrians were programmed to reach the participant’s lane edge in 5 seconds. In city drives, those at ±14° eccentricities moved faster (7-8mph) than those at ±4° eccentricities (3-4mph) as they had further to travel. On highways, pedestrians moved faster than in the city, at approximately 14–15 and 7–8 mph, for large and small eccentricities, similar to cycling and running speeds, respectively. Pedestrians on a collision course maintained a relatively constant eccentricity and thus stayed in the same area of the visual field for at least 3 s after appearance assuming participant's steady forward gaze towards the center of the travel lane [14, 19].

### Data Analyses

Dependent variables were detection rates, reaction times, and the proportion of untimely reactions. Reactions were “timely” if the horn press left sufficient time to brake and avoid colliding with the pedestrian hazard, had the pedestrian continued its course. We calculated the stopping distance of the vehicle from the time of horn press, assuming a deceleration rate of 5m/s^2^, representing dry level road and good tires [20].

We analyzed gaze data from pedestrian appearance to either the horn press, or to when the pedestrian disappeared (if undetected). To determine whether scotomas delayed reaction times, we computed the time the scotoma occluded the pedestrian during this period. Scotomas were modeled as convex polygons of ≤100 vertices. We converted binocular scotoma field plots measured with our custom system [17] from pixels to visual angles. Multiple scotomas (subject CFL5) were modeled as separate polygons. The pedestrian’s height was modeled as a vertical line segment at each sample (30Hz). When the pedestrian and scotoma overlapped horizontally, the percent of the pedestrian’s vertical extent inside the scotoma was calculated. Samples in which any part of the pedestrian was inside the scotoma were coded “occluded”.

We have no gaze-tracking data for one individual (CFL1). For all other participants we have gaze-tracking data for a median of 32 pedestrians per participant. Thus we had usable data for 37% (260) of all pedestrian appearances. We examined whether data dropouts induced any systematic bias in the distributions of pedestrian hazards with usable gaze data. We found that numbers of hazards were similar across all eccentricities (-14° n = 66/178; -4° n = 67/179, 4° n = 62/175, 14° n = 65/173), χ^2^ = 0.13, *p* = 0.99. There was no significant deviation from a uniform distribution for any individual participant (all *p*’s > 0.69), thus in gaze analyses, each hazard location was sampled approximately equally.

## Results

All participants had ≥15 years driving experience. Six CFL participants currently drove and one (CFL1) stopped 2 years previously after realizing that road signs were difficult to read. Of those currently driving, acuity and field extent were sufficient for two to be licensed with no restrictions (CFL3, CFL5) and one to be licensed with a daylight only restriction (CFL4) in Massachusetts. Current drivers completed the driving habits questionnaire [21]. Some CFL drivers mentioned avoiding difficult driving situations but overall reported driving more frequently than our controls (possibly because they lived further from Boston in areas less served by public transportation) (Table 2). NV controls likely drove much less due to their more convenient access to public transportation (only 1 reported driving to work).

**Table 2:** Driving Habits Questionnaire summary for current drivers.

Driving Habits	Central Field Loss (CFL) (n = 6)	Normal Vision (NV) (n = 7)
Someone in past year suggested limit driving, n	2	1
Self-rated driving quality, scale 1(worst)-5(best), median [range]	4.5 [2–5]	4 [4–5]
Avoided driving circumstances (n):		
parallel parking	3	1
highways, rush hour, high traffic	2	1
driving at night	2	0
Crash in past year, n	3	1
Number of days driving/week, median [range]	5 [5–7]	1 [0.5–7]
Miles driven/week, median [range]	71 [10–222]	17.8 [10–100]
Distance from Boston, median (miles) [range]	14 [6–35]	6 (2.5–93]

### Detection

Both CFL and NV drivers’ pedestrian detection rates were high (≥ 94%), however CFL1 detected <90% of pedestrians on the right. (Fig 1).

### Reaction Times

**Fig 2:**
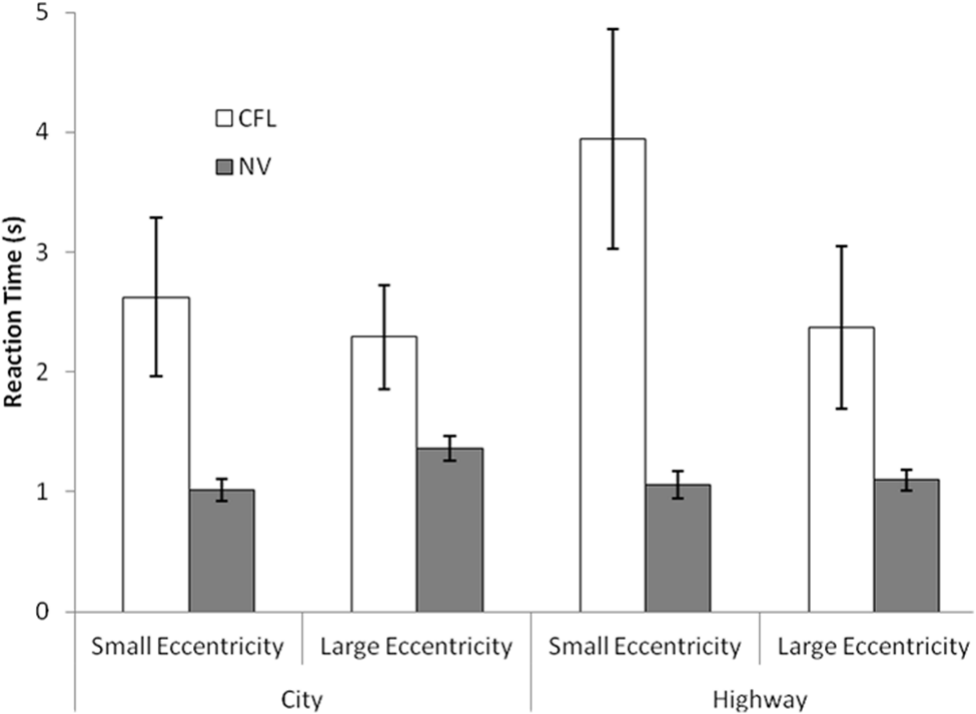
Central Field Loss (CFL) and Normal Vision (NV) participant mean reaction times by drive type and hazard eccentricity. CFL participants had longer reaction times than controls at all eccentricities. Error bars represent 95% confidence interval.

Median reaction times (RT) were calculated for each participant and eccentricity because RTs were non-normal. Medians were analyzed by repeated measures analysis of variance (ANOVA) with eccentricity (small/large) and drive type (city/highway) as within-subjects factors, and vision (NV/CFL) as a between-subjects factor. CFL participants’ RTs were longer than NV participants, overall (2.8s vs. 1.1s, F(1, 12) = 6.1, *p* = 0.03), and at each eccentricity in both drive types (Fig 2). CFL participants were slower in detecting pedestrians at the smaller vs. larger eccentricities (2.95s vs. 2.25s). Control participants were slower for the larger eccentricities (1.03s vs. 1.26s) but these differences were not significant *p* = 0.6. There was an interaction of eccentricity by vision, F (1,12) = 13.85, *p* = 0.003. CFL participants reacted more slowly to pedestrians on highway drives (3.16s vs. 2.46s city, *p* = 0.05), but NVs did not (1.08s vs. 1.19s). NVs were slower at large eccentricities on city drives (1.4s vs. 1.04s), whereas CFL participants were slower to small eccentricity pedestrians on highway drives (4.0s vs. 2.45s) (Fig 2) (3 way interaction of drive type by eccentricity and vision, F(1,12) = 5.15, *p* = 0.04).

### Gaze-Tracking Analyses

Pedestrians were partially or completely occluded by the scotoma prior to the horn press for 108 (42%) of the 260 appearances that have useable gaze data; an accounting of available data are provided in S2 Table. We expected and found that longer occlusion times correlated with longer response latencies (*r*(260) = 0.52, *p*<0.001).

**Fig 3:**
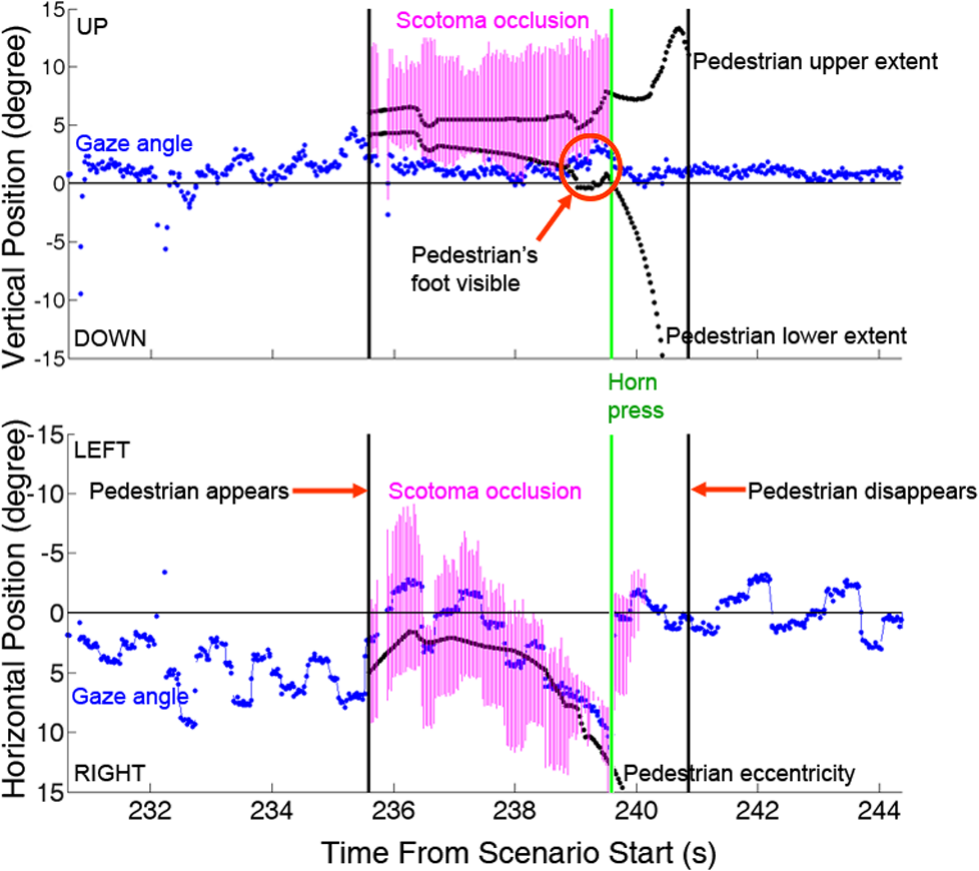
Example of a late pedestrian detection event in which the participant’s scotoma (Central Field Loss subject #4) was implicated. The upper graph depicts the pedestrian’s vertical position and size (black dots), the vertical extent and position of the scotoma at the horizontal position (magenta vertical lines), and the location of the vertical gaze Preferred Retinal Location (PRL) position (blue dots). Lower graph depicts horizontal position and gaze and the scotoma’s horizontal extent. When the pedestrian’s upper and lower limits are both within the vertical scotoma the pedestrian is not visible to the participant. Both the horizontal position of the pedestrian and the horizontal position and extent of the scotoma are relative to the car heading.

Fig 3 illustrates one example of a late pedestrian detection. When the pedestrian is occluded by the horizontal extent of the scotoma (lower panel) a portion protrudes beneath the scotoma at 239s (upper panel). The pedestrian may have been detected peripherally (but not identified) at about 236s, evidenced by horizontal rightward saccades at 236.5s and 238s before fixation at 238.5s and smooth tracking until 240s, culminating with a horn press near 239.8s. Because our perimetry may overestimate scotoma size, the pedestrian was likely detected near the scotoma’s lower edge. Note that when smooth pursuit is initiated (238.5s), indicating tracking of the pedestrian (lower horizontal graph), gaze is elevated (upper vertical graph) moving the pedestrian into the scotoma’s lower portion which is narrower horizontally (CFL4 Fig 1).

We used multiple linear regression to determine whether pedestrian occlusion time (from gaze-tracking data) and other vision and demographic measures predicted log-reaction times for each pedestrian appearance. Reaction times were log transformed as they were not normally distributed. This analysis is only of the CFL participants, and only covers the 37% of pedestrian appearances for which we have usable gaze-tracking data.

Longer reaction times were predicted by greater occlusion time and individual subject differences, overall model, *F*(3, 257) = 80.26, *p*<0.001, adjusted R^2^ = 0.38 (more details in S1 Table). Although scotoma size was correlated with reaction time (*r* = 0.58 (n = 7), *ns*), it did not improve model fit (no other vision measures did either). Most participants had strong positive correlations between reaction time and the amount of time their scotomas obscured pedestrians (Fig 1).

### Untimely Reaction Analysis

Logistic regression was conducted in SPSS v.11.5 using backward stepwise entry conditioned on the Wald statistic. CFL reaction times were sufficiently long that they would have collided with more pedestrians than NV controls (21% vs 3%), had pedestrians continued on their collision course (Wald = 32.22, df = 1, *p<*0.001, Exp(B) = 0.22). CFL participants had a higher proportion of untimely reactions to pedestrians at smaller (+/-4°) than larger eccentricities (24% vs 17%) Wald = 19.89, df = 1, *p <* 0.001, Exp(B) = 0.27; (S1 Fig), indicating an effect of the central scotoma. Both CFL and NV had more untimely reactions to pedestrians on highway drives than city drives (21% vs 8%) Wald = 55.40, df = 1, *p<*0.001, Exp(B) = 0.27).

Of the 39 untimely reactions that have gaze tracking data, 29 (74%) had some scotoma occlusion, whereas for timely reactions only 79/223 (35%) had occlusion (χ2 = 20.8 *p*<0.001). Untimely reactions were occluded, on average, 1.2 seconds (95%CI 0.7–1.7s) whereas timely reactions were occluded 0.3s (95%CI 0.2–0.4), *t*(40.5) = 3.8, *p*<0.001.

## Discussion

Because scotomas were usually above or below the gaze point on the roadway, and hazards were generally to the left or right of gaze, we predicted that responses would not vary with respect to lateral location of pedestrian hazards. Gaze-tracking, however, revealed that, due to vertical eye movements, scotomas occasionally obscured pedestrians, delaying reaction times (RTs). CFL participants were delayed enough that they would not have been able to stop for 21% of the pedestrians, seven times more than NV participants, even though pedestrians appeared at twice the recommended perception-brake sight distance (2x2.5s travel time) [22].

CFL participants responded later to pedestrians than NV controls (overall 2.30s vs. 1.06s), and their latencies were similar to lateral CFL participants in our prior study (2.46s) [14]. Removing the sole participant who was not a current driver did not change the results substantially or their statistical significance. Whereas vertical CFL response times were elevated for pedestrians more likely to appear near the scotoma (small vs. large eccentricity) (2.95s vs. 2.25s, respectively), lateral CFL participants in our prior study showed considerably later reactions for pedestrians near scotoma than non-scotoma locations (4.3s vs. 2.4s, respectively) [14]. We conducted a post-hoc repeated measures ANOVA comparing participants with lateral and vertical CFL. We hypothesized that participants with lateral CFL were slower to react to pedestrians than those with vertical scotomas, but only in areas more likely to be obscured by scotomas. That is, for those with vertical CFL the small (±4°) pedestrian eccentricities reaction times were longer than the large ±14° eccentricities. There was little difference in RTs between lateral and vertical CFLs in areas of the visual field unlikely to be obscured by scotomas (estimated marginal means both 2.4 s); however, lateral CFL response times were slower for pedestrians more likely to be obscured than for vertical CFL at the near eccentricity (4.3 vs. 3.3s), supported by an interaction of location (obscured or not obscured) by CFL group, *F*(1, 3.6) = 5.472, *p* = 0.03 (see S2 Fig).

These results support our main hypothesis that a vertical scotoma has less impact on hazard detection than a lateral scotoma; however, there appears to be increased risk of collision with scotomas in any configuration. Consistent with our prior study, control participants had slightly longer RTs to pedestrians at larger than at smaller eccentricities, likely due to reduced sensitivity in peripheral retina. Regression analysis indicated, for the vertical CFL participants, the amount of time that the scotoma occluded the pedestrian, even in part, was the most important predictor of RTs, and individual variation the next most important.

In our study of lateral scotomas, a smaller proportion of CFL participants were current drivers (3/11 vs. 6/7),[14] χ^2^(2) = 5.84, *p* = 0.02. However, they did not differ (t-test *p*s>0.26) from the current study population in age, visual acuity, contrast sensitivity, scotoma size, years of visual impairment, nor distance from Boston (those more distant are less likely to rely on public transportation).

We had usable gaze data for 37% of all pedestrian appearances in part because gaze tracking of participants with CFL is quite challenging due to unstable extra-foveal fixation, frequent use of eyeglasses, and advanced subject age. Thus, to our knowledge this is the first study using gaze tracking of CFL drivers in a realistic hazard detection task. Although the patient sample size was relatively small and diverse with respect to diagnosis, the number of pedestrian events per subject, combined with our within-subjects analyses, gave ample power to show reaction time deficits for the seven CFL drivers, six of whom were current and licensed drivers and **all had vision sufficient for a restricted license in at least some U.S. states** [23]. Participants with acuities worse than 0.55 LogMAR (CFL 2, 6, 7) may only be licensed to drive if they also use bioptic telescopes in Massachusetts; such telescopes are used only intermittently and briefly to inspect details, and it would be highly impractical to use one to scan for pedestrian or other road hazards[24]. Moreover, these results are consistent with our earlier findings on the impact of central and paracentral scotoma [14, 25]. Gaze tracking data revealed that a scotoma above or below the PRL may temporarily occlude hazards while driving. This might be due to intermittent examination of the dashboard/speedometer and/or eccentric fixation with the PRL being occasionally disrupted by “foveal” fixation [26]. In other words, a hazard could be perceived peripherally, but when a participant tries to confirm whether it is a threat, attempted fixation with the former fovea would bring the hazard into the scotoma. Such a phenomenon could cause a delayed response.

CFL participant RTs were also longer than NV for pedestrians not occluded by the scotoma. Reasons for this may include reduced contrast sensitivity due to increased retinal eccentricity and reduced attentional resources due to the effort required to maintain eccentric fixation with the PRL [27, 28]. Our simulator studies provide a specific mechanism by which AMD may cause difficulty in driving and perhaps cessation altogether. Taken together, the results of our studies suggest that presence of binocular CFL, regardless of PRL location or etiology, might cause delayed responses to hazards on the road and should be considered when evaluating vision for driving, either by practitioners who advise their patients on driving with low vision or by state licensing agencies.

## Supporting Information

S1 Fig Individual proportion of untimely responses for pedestrians at small and large eccentricities. Data for each participant with Central Field Loss (CFL) and each Normal Vision (NV) participant are connected by black and grey straight lines, respectively. In general, participants with CFL had much higher untimely response rates than NV controls, particularly for pedestrians at small eccentricities and on rural highways. Controls also had more untimely reactions in rural highway than city drives.
(TIF)

S2 Fig Mean reaction times for vertical central field loss (CFL) participants in this study and lateral CFL participants from the prior study [14]. Compared to those with vertical field loss, participants with lateral field loss had considerably longer reaction times to pedestrians likely to be obscured (“likely obscured” vs. ±4°), but similar reaction times to pedestrians not likely to be obscured (“seeing” vs. ±14°). Error bars represent 95% confidence interval.
(TIF)

S1 Table Multiple regression results. (DOCX)

S2 Table Gaze tracker data available for participants. (DOCX)
