# Supplementary figures and images for: Driving with Central Visual Field Loss II: How Scotomas above or below the Preferred Retinal Locus (PRL) Affect Hazard Detection in a Driving Simulator
Source: PLoS One. 2015 Sep 2;10(9):e0136517. doi: 10.1371/journal.pone.0136517 (PMC4557943; doi:10.1371/journal.pone.0136517)

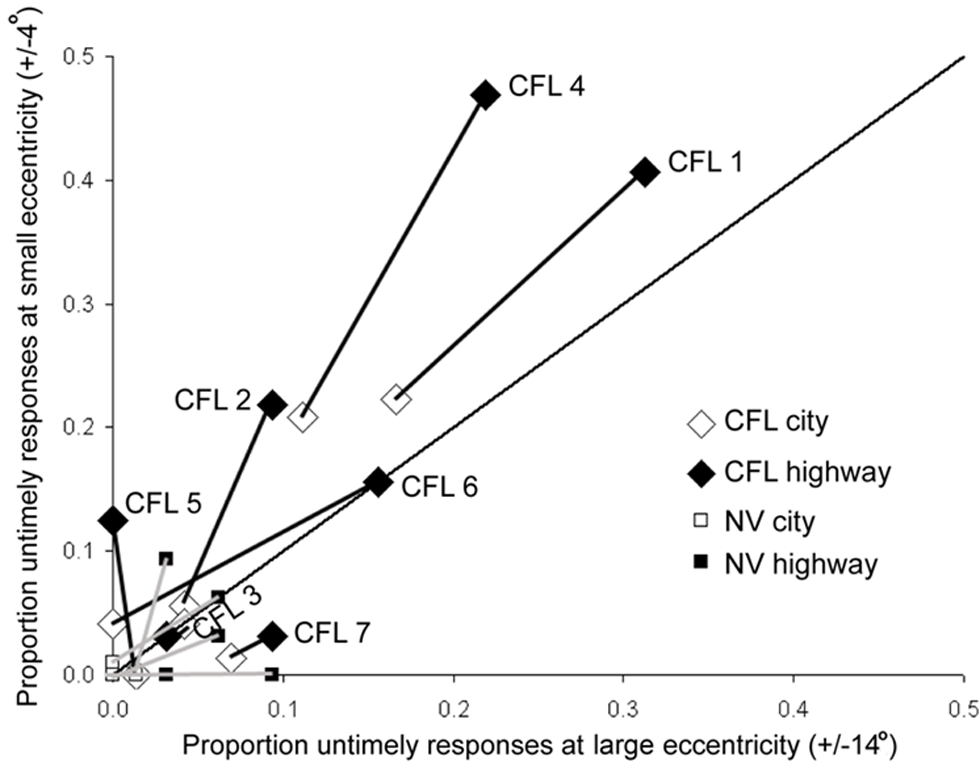

Supplement: S1 Fig — Data for each participant with Central Field Loss (CFL) and each Normal Vision (NV) participant are connected by black and grey straight lines, respectively. In general, participants with CFL had much higher untimely response rates than NV controls, particularly for pedestrians at small eccentricities and on rural highways. Controls also had more untimely reactions in rural highway than city drives. (TIF) [file pone.0136517.s001.tif]

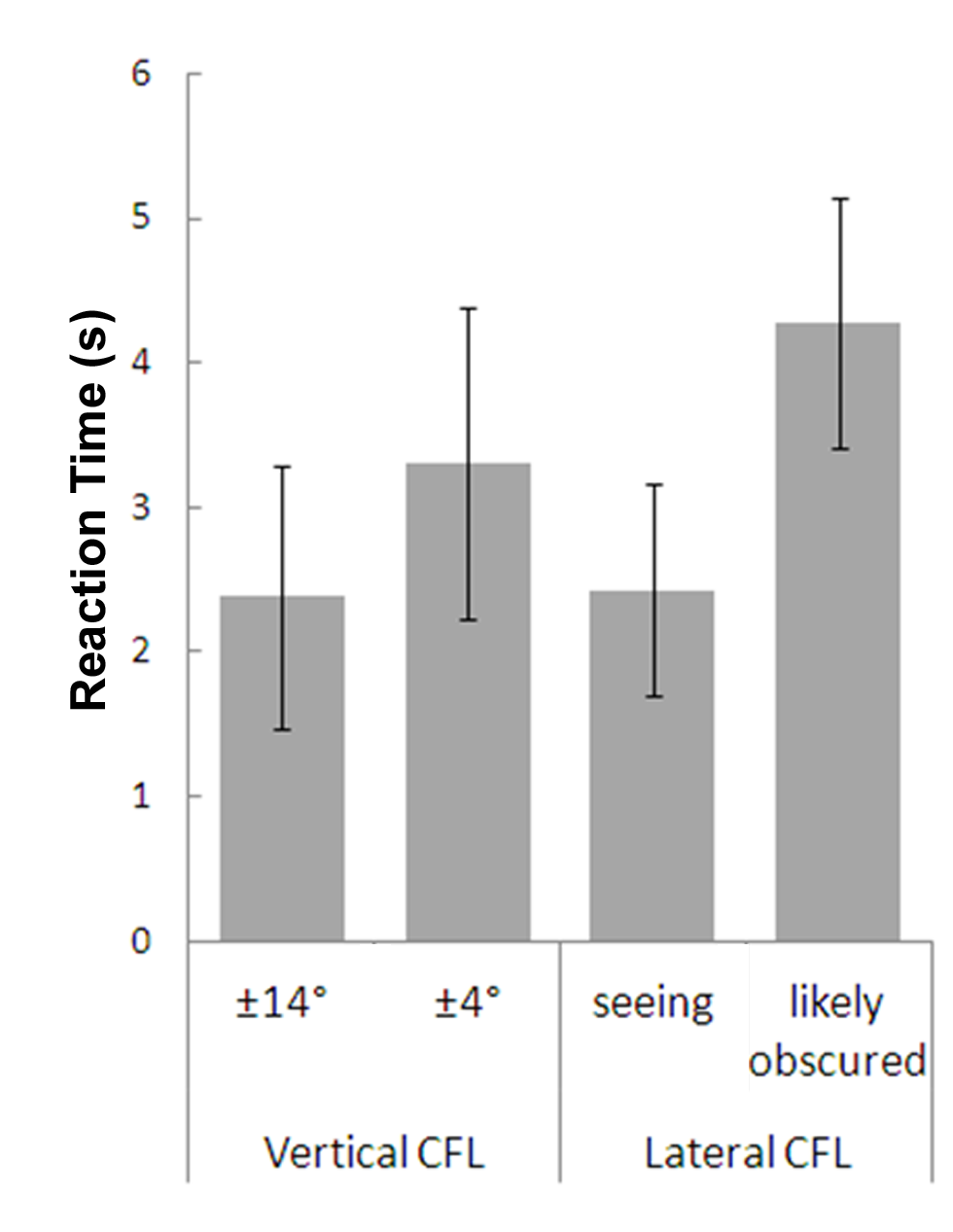

Supplement: S2 Fig — Compared to those with vertical field loss, participants with lateral field loss had considerably longer reaction times to pedestrians likely to be obscured (“likely obscured” vs. ±4°), but similar reaction times to pedestrians not likely to be obscured (“seeing” vs. ±14°). Error bars represent 95% confidence interval. (TIF) [file pone.0136517.s002.tif]
