# Supplementary material for: Driving with Central Visual Field Loss II: How Scotomas above or below the Preferred Retinal Locus (PRL) Affect Hazard Detection in a Driving Simulator
Source: PLoS One. 2015 Sep 2;10(9):e0136517. doi: 10.1371/journal.pone.0136517 (PMC4557943; doi:10.1371/journal.pone.0136517)
Supplement: S1 Table — (DOCX) [file pone.0136517.s003.docx]

Table S1. Multiple Regression Results.

|  | **Correlation with Log Reaction Time (RT)** | | | **Factor Statistics** | |
| --- | --- | --- | --- | --- | --- |
| **Factor** | **Zero^*^** | **Partial^**^** | **Part^***^** | **t** | **Sig** |
| Pedestrian obscured (s) | 0.52 | 0.33 | 0.28 | 5.69 | *p*<0.001 |
| Subject | 0.55 | 0.40 | 0.34 | 6.99 | *p*<0.001 |
| **Not in model** |  |  |  |  |  |
| Age | -0.18 |  | | | |
| Contrast sensitivity | -0.14 |  |  |  |  |
| Drive speed | 0.07 |  |  |  |  |
| Fixation stability | -0.08 |  |  |  |  |
| Scotoma size | 0.27 |  |  |  |  |
| Visual acuity | 0.04 |  |  |  |  |
| ^*^Zero: correlation of the individual factor with log RT without consideration of other factors; ^**^Partial: correlation after removing mutual association with other factors in the model; ^***^Part: correlation after removing linear effects of other factors from log RT. | | | | | |
