# Supplementary material for: Driving with Central Visual Field Loss II: How Scotomas above or below the Preferred Retinal Locus (PRL) Affect Hazard Detection in a Driving Simulator
Source: PLoS One. 2015 Sep 2;10(9):e0136517. doi: 10.1371/journal.pone.0136517 (PMC4557943; doi:10.1371/journal.pone.0136517)
Supplement: S2 Table — (DOCX) [file pone.0136517.s004.docx]

**Table S2.** Gaze tracker data available for participants.

| **Participant** | Pedestrians  Detected (n) | % with  Gaze Data | Cause of data loss |
| --- | --- | --- | --- |
| CFL1 | 97 | 0% | Gaze not tracked; session preceded eye-tracker implementation |
| CFL2 | 103 | 22% | Spectacle lenses caused IR reflections to often obscure eyes |
| CFL3 | 101 | 92% | No systematic cause indicated |
| CFL4 | 102 | 69% | Posture change between drives |
| CFL5 | 104 | 39% | Spectacle lens reflections |
| CFL6 | 104 | 19% | Short stature caused hand, wheel to occlude face from the gaze tracker cameras |
| CFL7 | 96 | 14% | Unknown / optical correction |
